# Neuromedin U Suppresses Collagen-Induced Arthritis through ILC2-Th2 Activation

**DOI:** 10.1155/2021/5599439

**Published:** 2021-03-08

**Authors:** Yuanyuan Zhang, Yi Qin, Zhu Chen

**Affiliations:** Department of Rheumatology and Immunology, The First Affiliated Hospital of USTC, Division of Life Sciences and Medicine, University of Science and Technology of China, Lujiang Str. 17, Hefei 230001, China

## Abstract

Neuromedin U (NMU) is an evolutionarily conserved neuropeptide which was previously thought to have a proinflammatory property. Recently, it was reported that NMU induced rapid ILC2 activation and Th2 responses in allergic diseases. However, whether NMU could launch such responses in arthritis is not known. In this study, we investigated the effect of NMU administration on arthritis and its underlying mechanisms. C57BL/6 male mice were induced with collagen-induced arthritis (CIA) and treated with NMU-23 or PBS at an early stage of induction. NMU-23 dramatically inhibited clinical onset and severity of arthritis, accompanied with decreased bone erosion and number of osteoclasts. Mechanistically, NMU-23 administration induced the expansion of ILC2 and elevated eosinophil, IL-5, and IL-13 expression in the joint of arthritic mice. Although levels of Th2 cells are slightly increased, Gata3 expression level is also upregulated. Further, NMU-deficient (NMU^−/−^) mice develop less severe CIA compared with control. Interestingly, the proportion of ILC2 and FoxP3^+^ regulatory T cells (Treg) was elevated in NMU^−/−^ mice. Taken together, our results reveal a previously unknown anti-inflammatory effect of NMU in CIA by inducing ILC2-Th2 activation.

## 1. Introduction

Neuromedin U (NMU) is an evolutionarily conserved neuropeptide initially isolated from porcine spinal cord [1]. Although named for its ability to induce uterine smooth muscle contraction, NMU has been reported to have roles in metabolic regulation, blood pressure control, bone mass homeostasis, and pain sensations [2]. Interestingly, increasing evidence has emerged that NMU also participates in immunological processes. One early study demonstrated that exposure of a mouse Th2 cell clone to NMU led to intracellular calcium flux and release of Th2 cytokines like IL-4 and IL-13 [3]. More recently, three independent groups found that ILC2 selectively expresses the NMU receptor 1 (NMUR1). Stimulation of ILC2 with NMU induced more rapid ILC2 activation and secretion of Th2 cytokines IL-5, IL-9, and IL-13, compared with epithelial cell-derived alarmin cytokines such as IL-25 and IL-33 [4–6]. These findings suggest that NMU might be an important regulator of type 2 immune responses.

Innate lymphoid cells (ILCs) have recently been identified as a family of innate immune systems characterized by the absence of lineage markers. Serving as tissue resident “sentinel” cells, ILCs are considered as first responders of tissue damage signals, launching critical immune responses before adaptive immunity starts [7]. Actually, ILCs have been observed to be involved in the pathogenesis of many autoimmune and inflammatory diseases, including systemic lupus erythematosus (SLE), rheumatoid arthritis (RA), and spondyloarthritis [8–12]. In contrast with the proinflammatory role of ILC1 and ILC3 in RA, ILC2 has been considered to exert an anti-inflammatory effect. Genetic deletion of ILC2 leads to aggravated arthritis, whereas activation of ILC2 induces resolution of arthritis in a mouse model [9]. Furthermore, RA patients in remission have higher peripheral ILC2 than active patients [10]. Considering that ILC2 was linked to Th2 activation, which has recently been proved as a regulatory pathway in RA by us [13], regulation of ILC2 might be a promising therapeutic strategy in arthritis treatment. Hereby, we utilized a collagen-induced arthritis (CIA) model to test the hypothesis that NMU suppresses arthritis through ILC2-Th2 activation. We further investigated the effect of NMU deletion in the development of CIA.

## 2. Materials and Methods

### 2.1. Mice

C57BL/6 mice were purchased from Changzhou Cavins Laboratory Animal Co., Ltd. (Suzhou, China) and maintained in specific pathogen-free (SPF) facilities. Mice with an NMU deletion (NMU^−/−^) on a C57BL/6 background have been described before and were purchased from Cyagen (Suzhou, China) [14]. Only male mice aged from 8 to 12 weeks were used for the induction of arthritis. This study was approved by the Animal Ethics Committee of the First Affiliated Hospital of the University of Science and Technology of China. All animal experiments were performed in accordance with the National Institutes of Health “Guide for the Care and Use of Laboratory Animals”.

### 2.2. Induction of Collagen-Induced Arthritis (CIA)

CIA was established as previously reported [15]. NMU-23, the rodent isoform of the NMU, was purchased from Phoenix Pharmaceuticals as previously reported [5, 6]. Eight C57BL/6 male mice were treated i.p. daily by 20 *μ*g NMU-23 or PBS for 10 days from day 1 to 5 and from day 21 to 25 during induction of CIA. The clinical severity of CIA was assessed every two days starting on day 21 using a previously established macroscopic scoring system [15].

### 2.3. Histology

Arthritic mice were sacrificed on day 42. Knee joints and hind paws were removed and fixed in 4% paraformaldehyde overnight, decalcified for 14 days, and embedded into paraffin blocks, which were then sliced into 5 mm thick sections and sent for hematoxylin and eosin (H&E) and tartrate-resistant acid phosphatase (TRAP) staining. The procedure to evaluate the joint inflammation and bone erosion was described previously [13].

### 2.4. Flow Cytometry

Spleen, mesenteric lymph node (mLN), and ankle joints were processed for flow cytometry as we previously described [13]. The following antibodies were used for surface molecular staining: BV510-anti-CD45 (30F11), FITC-CD4 (JK1.5), FITC-Lineage (CD3/GR-1/CD11b/CD45R/TER-119), BV421-CD127 (A7R34), PerCP/Cy5.5-KLRG1 (2F1), PE-ST2 (RMST2-2), APC-ICOS (C398.4A), APC-NK1.1 (PK136), PerCP/Cy5.5-Ly6G(1A8), FITC-CD11b(M1/70), APC-F4/80 (BM8, all from BioLegend), and PE-SiglecF (1RNM44N, eBioscience). For intracellular staining, cells were fixed and permeabilized by the FoxP3/Transcription Factor Staining Buffer (eBioscience) and then stained with PE/Cy7-anti-T-bet (4B10), PE-anti-ROR*γ*t (AFKGS-9), PerCP/Cy5.5-IFN-*γ* (XMG1.2, BD Biosciences), APC-IL-4 (11B11, BioLegend), and AF647-IL-17A (TC11-18H10, BD Biosciences) at 4°C for 30 min. In some experiments, mLN cells and splenocytes were plated on a 12-well plate and incubated with cell activation cocktail (with Brefeldin A, BioLegend) at 37°C for 4 h before staining.

### 2.5. Quantitative RT-PCR

Total RNA was extracted from the spleen and joints using the HiPure Total RNA Mini Kit (Magen, China) according to the manufacturer's instructions and reverse transcribed to cDNA with the FastKing gDNA Dispelling RT Supermix (Tiangen, China). Quantitative RT-PCR was performed using the QuantiNova SYBR Green PCR Kit (QIAGEN, Germany) on the QuantStudio Q5 real-time PCR system. GAPDH was used as an internal control. Relative gene expression was calculated by the 2^∆∆Ct^ method. All the primer sequences are listed in Supplementary Table 1.

### 2.6. Luminex Analyses

Cytokine (TNF-*α*, IL-4, IL-5, and IL-6) levels in plasma from CIA mice treated with PBS or NMU-23 were measured by the Luminex Mouse Magnetic Assay (R&D Systems) according to the manufacturer's instructions. All measurements were performed with Luminex 200™ device (Luminex Corporation, USA).

### 2.7. Statistical Analyses

All data were presented as mean ± SEM. Comparison between groups were analyzed using unpaired Student's *t*-test or Mann–Whitney test. *P* < 0.05 was regarded as statistically significant.

## 3. Results

### 3.1. NMU-23 Dramatically Inhibited Clinical Onset and Severity of Arthritis in CIA Mice

**Figure 1:**
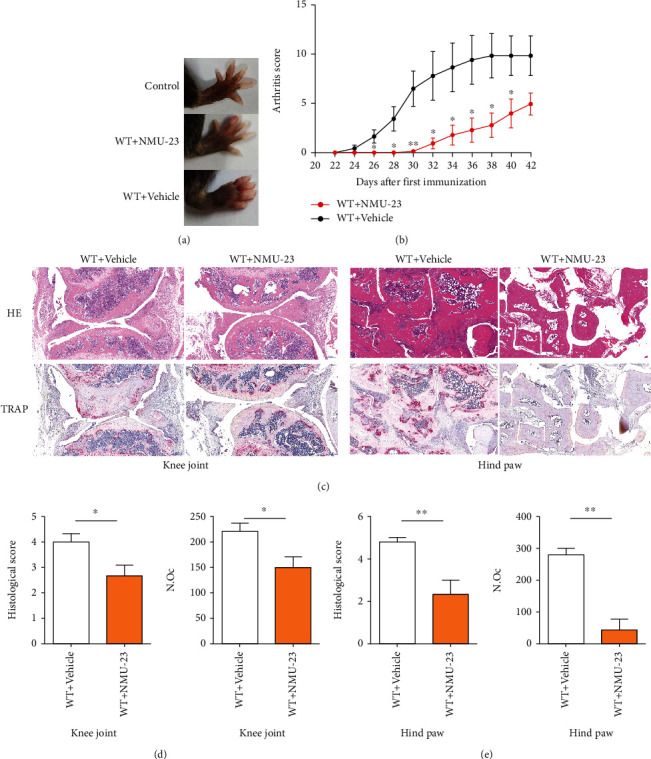
NMU-23 inhibited joint inflammation and bone erosion in CIA mice. (a) Representative hind paw of mice from WT control (*n* = 3), WT treated with NMU-23 (*n* = 8), and WT treated with PBS (*n* = 8). (b) Clinical assessment of CIA mice determined every 2 days (*n* = 8 each in two independent experiments). (c) Representative hematoxylin-eosin (H&E) and tartrate-resistant acid phosphatase (TRAP) staining of the knee joint and hind paw of mice. (d, e) Quantification of histological score and number of osteoclasts in the knee joint (d) and hind paw (e) of mice. Data are shown as mean ± SEM (^∗^*P* < 0.05 and ^∗∗^*P* < 0.01 determined by Student's *t*-test).

We first investigated the effects of NMU-23 on the development of CIA. Eight C57BL/6 male mice were treated i.p. daily with 20 *μ*g NMU-23 or PBS from days 1 to 5 and from days 21 to day 25 during CIA induction. As shown in Figures 1(a) and 1(b) and Supplemental Figures 1a–1b, NMU-23 dramatically inhibited clinical onset and arthritis score of arthritis in the treated mice, compared with control mice. In addition, histological examination on day 42 also revealed a significant decrease of joint inflammation, bone erosion, and the number of osteoclasts in NMU-23-treated mice (Figures 1(c)–1(e)). Thus, our results indicate that arthritis development and severity were significantly alleviated by NMU-23 administration.

### 3.2. Expansion of ILC2 in Arthritic Joint of Mice Treated with NMU-23

**Figure 2:**
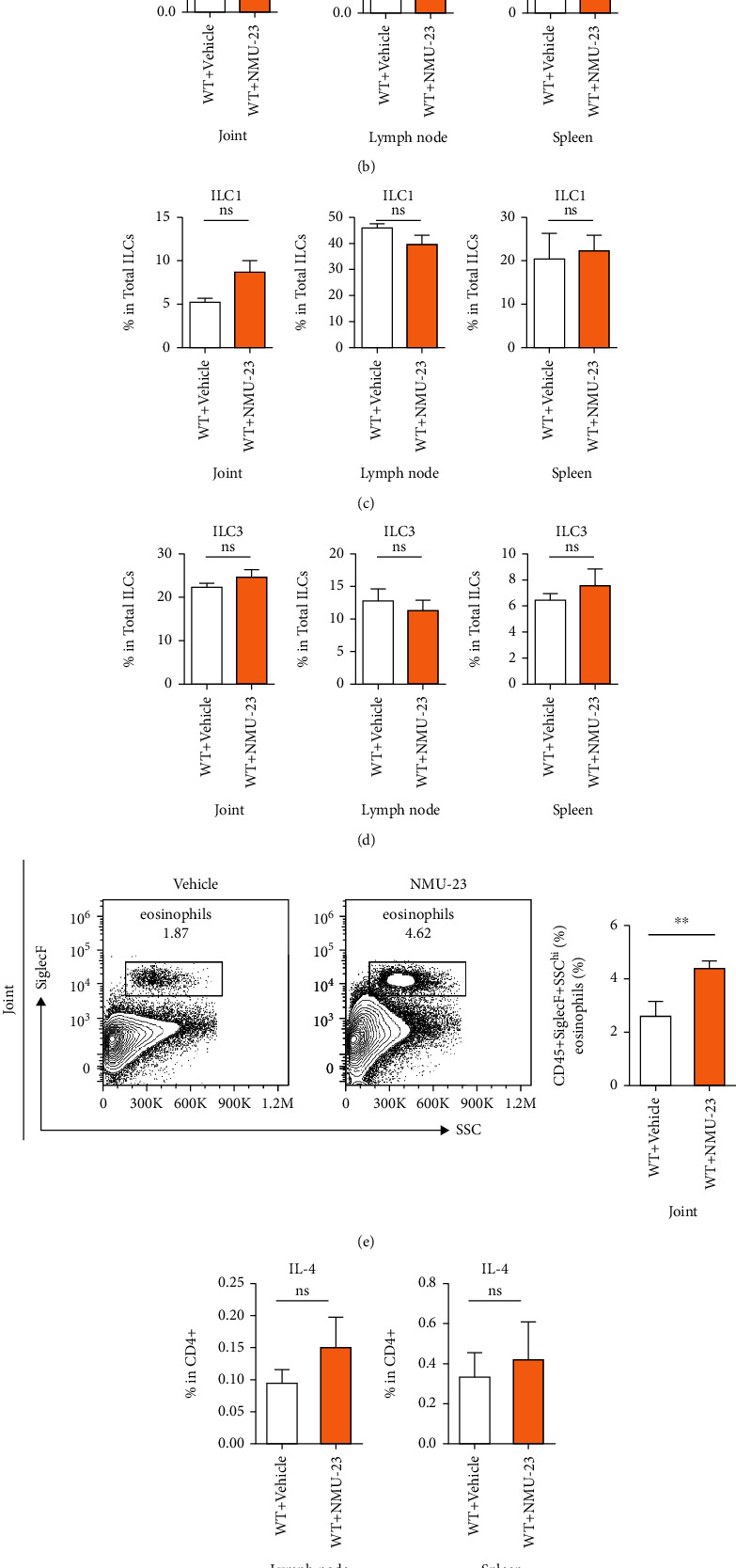
NMU-23 induced ILC2 expansion and Th2 responses in the joint of arthritic mice. (a) Gating strategy for the identification of total ILCs (CD45^+^Lineage^−^CD127^+^), ILC1 (NK1.1^+^T-bet^+^), ILC2 (KLRG1^+^ICOS^+^), and ILC3 (NK1.1^−^ROR*γ*t^+^) in mesenteric lymph node (mLN) of arthritic mice. (b–d) The frequency of ILC2 (b), ILC1 (c), and ILC3 (d) in the joint, mLN, and spleen of arthritic mice. (e) Representative flow cytometry plot and quantification of eosinophils (CD45^+^SiglecF^+^) in the joint of arthritic mice. (f) The percentage of Th2 cells in mLN and spleen of arthritic mice. (g) Sera TNF-*α*, IL-4, IL-5, and IL-6 levels were determined by Luminex. Data are shown as mean ± SEM (^∗^*P* < 0.05, ^∗∗^*P* < 0.01, and ^∗∗∗^*P* < 0.001 determined by Student's *t*-test).

It has been reported that NMU induced ILC2 activation and amplified allergic lung inflammation [4]. It is not known, however, whether NMU can initiate such innate type 2 immune responses in arthritis. To test the hypothesis that the inhibitory effect of NMU-23 on CIA involved ILC2 activation, we analyzed ILC populations in the joint, spleen, and mLN by flow cytometry (Figure 2(a)). As expected, we found that the proportion of ILC2 (defined as CD45^+^Lin^−^ CD127^+^ICOS^+^ST2^+^) was elevated in the joint of NMU-23-treated mice as compared to control mice (Figure 2(b) and Supplemental Figure 1c). However, we did not find increased ILC2 in the spleen and mLN on day 42, which might suggest that local expansion of ILC2 induced by NMU-23 was responsible for the reduction of arthritis development (Figure 2(b)). In contrast, the proportion of ILC1 (defined as CD45^+^Lin^−^ CD127^+^NK1.1^+^T-bet^+^) and ILC3 (defined as CD45^+^Lin^−^CD127^+^NK1.1^−^ROR*γ*t^+^) was not changed between the two groups (Figures 2(c) and 2(d)).

### 3.3. NMU-23 Promoted Th2 Immune Responses in Arthritic Mice

We have recently shown that eosinophils and Th2 activation suppress arthritis [13, 16]. Therefore, next we investigated the effect of NMU-23 on eosinophils and Th2 cells. First, NMU-23 administration induced a significant increase of eosinophils (CD45^+^SiglecF^+^) in the joint of arthritic mice (Figure 2(e)). Other cell types, including regulatory T cells (Treg), neutrophils, and macrophages, were not changed (Supplemental Figures 1d–1f). Next, we examined the proportion of Th2 cells, defined as CD4^+^IL-4^+^ cells, in the spleen and mLN of arthritic mice. The frequency of Th2 cells was slightly increased in the spleen of NMU-2-treated mice, but there is no statistical significance (Figure 2(f)). Interestingly, the proportion of CD4^+^IFN-*γ*^+^Th1 cells was increased in mLN, whereas Th17 cells were comparable between two groups (Supplemental Figures 1g–1h).

**Figure 3:**
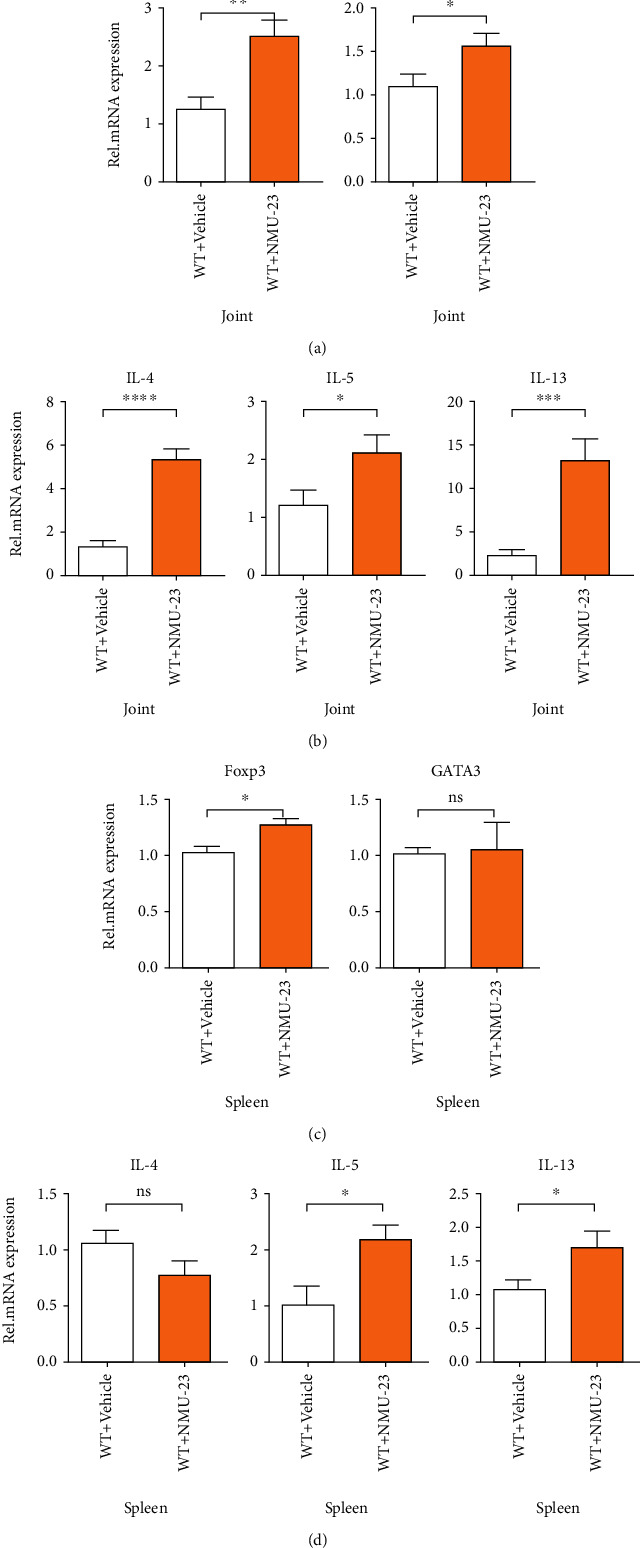
Th2-related cytokines and nuclear transcription factor mRNA expression levels were upregulated by NMU-23 treatment. (a, b) mRNA expression of FoxP3, Gata3 (a), IL-4, IL-5, and IL-13 (b) in the joint of vehicle- and NMU-23-treated mice. (c, d) mRNA expression of FoxP3, Gata3 (c), IL-4, IL-5, and IL-13 (d) in the spleen of vehicle and NMU-23-treated mice. Data are shown as mean ± SEM (^∗^*P* < 0.05, ^∗∗^*P* < 0.01, ^∗∗∗^*P* < 0.001, and ^∗∗∗∗^*P* < 0.0001 determined by Student's *t*-test).

Further, we assessed Th1/Th2-related cytokines in the sera and found that IL-5 levels were significantly elevated after NMU-23 treatment (Figure 2(g)). In addition, mRNA expression levels of Th2-associated cytokines and nuclear transcription factors were analyzed in the joint and spleen of CIA mice. As shown in Figures 3(a) and 3(c), the mRNA expression levels of *Gata3* were increased in the joint but not in the spleen of NMU-23-treated CIA mice. *Il5* and *Il13*, which are markers of ILC2-Th2 activation, were significantly upregulated both in the joint and in the spleen of NMU-23-treated CIA mice (Figures 3(b) and 3(d)). *Il4* mRNA expression was also elevated in the joint of NMU-23-treated mice (Figures 3(b) and 3(d)). Interestingly, the increased expression of FoxP3 in the joint and spleen was also noted (Figures 3(a) and 3(c)). Taken together, these results suggest that NMU-23 induced global Th2 immune responses in the joint of arthritic mice.

### 3.4. NMU-Deficient Mice Develop Less Severe CIA

**Figure 4:**
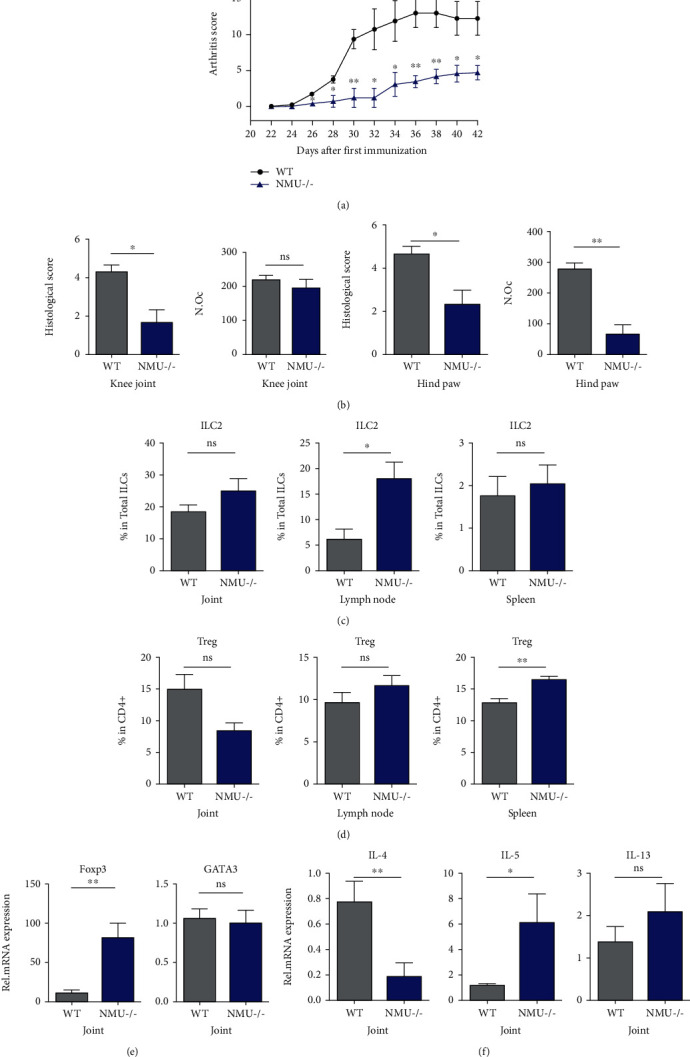
NMU-deficient mice developed significantly less severe arthritis. (a) Clinical assessment of NMU^+/+^ and NMU^−/−^ CIA mice (*n* = 4 each in two independent experiments) determined every 2 days. (b) Quantification of histological score and number of osteoclasts in the knee joint and hind paw of mice. (c) The frequency of ILC2 in the joint, mLN, and spleen of arthritic mice. (d) The frequency of Treg in the joint, mLN, and spleen of arthritic mice. (e, f) mRNA expression of FoxP3, Gata3 (e), IL-4, IL-5, and IL-13 (f) in the joint of WT and NMU^−/−^ mice. Data are shown as mean ± SEM (^∗^*P* < 0.05 and ^∗∗^*P* < 0.01 determined by Student's *t*-test).

It has been reported before that NMU-deficient mice are protected from developing autoantibody-induced arthritis [14]. Hereby, we are wondering about the effect of NMU deletion on CIA. NMU^−/−^ male mice on a C57BL/6 background were subject to induce CIA. Compared with NMU^+/+^ littermate control, NMU^−/−^ mice develop significantly less severe arthritis (Figure 4(a) and Supplemental Figures 2a and 2b). Accordingly, histological score and number of osteoclasts were lower in NMU^−/−^ mice (Figure 4(b) and Supplemental Figure 2c). Next we assessed ILC2 populations and found that the proportion of ILC2 was increased in mLN but not in the spleen and joints of NMU^−/−^ mice (Figure 4(c)). However, the proportion of Th2 and Th17 cells in the spleen and mLN was not changed between NMU^−/−^ mice and control, whereas the proportion of Th1 cells was higher in mLN of NMU^−/−^ mice (Supplemental Figure 2d). Interestingly, CD4^+^CD25^+^FoxP3^+^ Treg was also expanded in the spleen of NMU^−/−^ mice, which might contribute to the downregulated joint inflammation (Figure 4(d)). We further examined mRNA expression levels of Th2-related cytokines and nuclear transcription factors in the joint. Similar with NMU-23-treated mice, elevated *Il5* and *Foxp3* mRNA expression in the joint was observed in NMU^−/−^ mice (Figures 4(e) and 4(f)). Unexpectedly, *Il4* mRNA expression was downregulated in NMU^−/−^ mice (Figure 4(f)).

## 4. Discussion

NMU has been increasingly recognized as a potential regulator in autoimmune and inflammatory diseases [17]. NMU and NMUR1 mRNA have been detected in immune cells, including dendritic cells, monocytes, and T cells, suggesting a role of NMU-NMUR1 signaling in immune responses [18]. Previously, it has been shown that mice with NMU deletion have high bone mass due to an increase of bone formation [19]. Up to date, there is only one study reporting that NMU-deficient mice are protected from developing autoantibody-mediated arthritis [14]. However, the mechanisms behind and whether NMU itself could modify inflammatory arthritis are unclear.

**Figure 5:**
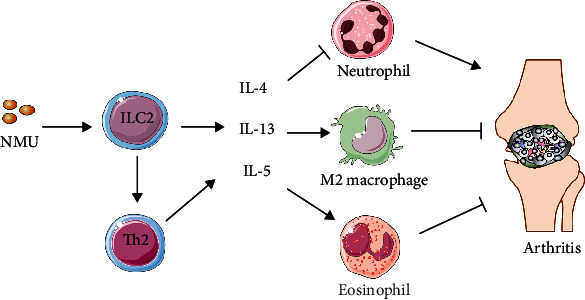
Mechanisms of suppression of arthritis induced by NMU. NMU stimulated ILC2 and subsequently activated Th2, leading to the production of IL-4, IL-13, and IL-5. These type 2 cytokines further activated eosinophils and anti-inflammatory M2 macrophages, together with decreased neutrophil activity, promoting resolution of arthritis.

Our study demonstrated that NMU administration at an early phase significantly inhibited the onset and severity of CIA for the first time. Mechanistically, NMU administration induced the activation of the ILC2-Th2 axis, as evidenced by the expansion of ILC2 and the elevated IL-5 and IL-13 expression levels in the joint of arthritic mice. In addition, mRNA expression of Th2-related nuclear factor Gata3 was also increased. This is consistent with recent studies, which have established that NMU amplified allergic lung inflammation by rapid induction of ILC2 and Th2 responses [4]. Indeed, early studies have shown that NMU could activate a variety of effector cells, such as eosinophils and macrophages, by inducing cell adhesion and chemotaxis toward inflammatory sites and promoting production of IL-6 [20, 21]. Although neutrophils and macrophages were not altered in our study, we revealed an increase of eosinophils after NMU administration, which have been shown to be a negative regulator of joint inflammation [13, 22]. These results indicate a novel anti-inflammatory role of NMU in arthritis. The general mechanisms of how NMU alleviates arthritis are illustrated in Figure 5.

To further investigate the effect of NMU in arthritis, we utilized NMU-deficient mice for the induction of CIA. Similar with a previous study in K/BxN serum-induced arthritis, mice with NMU deletion developed less CIA compared with control. Interestingly, we found elevated ILC2 levels in mLN of NMU^−/−^ mice, which have not been reported before [14]. Consistently, IL-5 mRNA expression level was upregulated in the joints of NMU^−/−^ mice. In addition, increased Treg levels were observed in the spleen of NMU^−/−^ mice, which might contribute to suppress arthritis. However, we did not find altered Th2 responses but elevated levels of Th1 cells with NMU deletion. This might be explained by the fact that NMU has been considered a proinflammatory neuropeptide [21]. The limitations of our study are the lack of molecular mechanisms downstream of ILC2-Th2 activation; also, whether NMU has the same effect in other types of arthritis remains unclear. Further in vitro studies and validation in other types of arthritis are needed in future work.

## 5. Conclusions

Taken together, our results suggest that NMU has a previously unknown anti-inflammatory effect in collagen-induced arthritis by inducing ILC2-Th2 activation.

## Data Availability

Data will be available from authors on request.

## Supplementary Materials

Supplementary Materials Supplemental Figure 1: clinical and immunological assessment of mice treated with NMU-23. Supplemental Figure 2: clinical and immunological assessment of NMU^+/+^ and NMU^−/−^ CIA mice. Supplemental Table 1: the sequences of primers for quantitative RT-PCR.

## References

[1] Gajjar S., Patel B. M. (2017). Neuromedin: an insight into its types, receptors and therapeutic opportunities. *Pharmacological Reports*.

[2] Martinez V. G., O'Driscoll L. (2015). Neuromedin U: a multifunctional neuropeptide with pleiotropic roles. *Clinical Chemistry*.

[3] Johnson E. N., Appelbaum E. R., Carptenter D. C. (2004). Neuromedin U elicits cytokine release in murine Th2-type T cell clone D10.G4.1. *Journal of Immunology*.

[4] Wallrapp A., Riesenfeld S. J., Burkett P. R. (2017). The neuropeptide NMU amplifies ILC2-driven allergic lung inflammation. *Nature*.

[5] Klose C. S. N., Mahlakõiv T., Moeller J. B. (2017). The neuropeptide neuromedin U stimulates innate lymphoid cells and type 2 inflammation. *Nature*.

[6] Cardoso V., Chesné J., Ribeiro H. (2017). Neuronal regulation of type 2 innate lymphoid cells via neuromedin U. *Nature*.

[7] Artis D., Spits H. (2015). The biology of innate lymphoid cells. *Nature*.

[8] Hou M., Liu S. (2019). Innate lymphoid cells are increased in systemic lupus erythematosus. *Clinical and Experimental Rheumatology*.

[9] Omata Y., Frech M., Primbs T. (2018). Group 2 innate lymphoid cells attenuate inflammatory arthritis and protect from bone destruction in mice. *Cell Reports*.

[10] Rauber S., Luber M., Weber S. (2017). Resolution of inflammation by interleukin-9-producing type 2 innate lymphoid cells. *Nature Medicine*.

[11] Soare A., Weber S., Maul L. (2018). Cutting edge: homeostasis of innate lymphoid cells is imbalanced in psoriatic arthritis. *Journal of Immunology*.

[12] Leijten E. F., van Kempen T. S., Boes M. (2015). Brief report: enrichment of activated group 3 innate lymphoid cells in psoriatic arthritis synovial fluid. *Arthritis & Rhematology*.

[13] Chen Z., Andreev D., Oeser K. (2016). Th2 and eosinophil responses suppress inflammatory arthritis. *Nature Communications*.

[14] Rao S. M., Auger J. L., Gaillard P. (2012). The neuropeptide neuromedin U promotes autoantibody-mediated arthritis. *Arthritis Research & Therapy*.

[15] Brand D. D., Latham K. A., Rosloniec E. F. (2007). Collagen-induced arthritis. *Nature Protocols*.

[16] Liu L., Zhang Y., Zheng X. (2019). Eosinophils attenuate arthritis by inducing M2 macrophage polarization via inhibiting the I*κ*B/P38 MAPK signaling pathway. *Biochemical and Biophysical Research Communications*.

[17] Ye Y., Liang Z., Xue L. (2021). Neuromedin U: potential roles in immunity and inflammation. *Immunology*.

[18] Hedrick J. A., Morse K., Shan L. (2000). Identification of a human gastrointestinal tract and immune system receptor for the peptide neuromedin U. *Molecular Pharmacology*.

[19] Sato S., Hanada R., Kimura A. (2007). Central control of bone remodeling by neuromedin U. *Nature Medicine*.

[20] Moriyama M., Fukuyama S., Inoue H. (2006). The neuropeptide neuromedin U activates eosinophils and is involved in allergen-induced eosinophilia. *American Journal of Physiology. Lung Cellular and Molecular Physiology*.

[21] Moriyama M., Matsukawa A., Kudoh S. (2006). The neuropeptide neuromedin U promotes IL-6 production from macrophages and endotoxin shock. *Biochemical and Biophysical Research Communications*.

[22] Andreev D., Liu M., Kachler K. (2020). Regulatory eosinophils induce the resolution of experimental arthritis and appear in remission state of human rheumatoid arthritis. *Annals of the Rheumatic Diseases on pages annrheumdis to 2020*.

