# Supplementary material for: Neuromedin U Suppresses Collagen-Induced Arthritis through ILC2-Th2 Activation
Source: J Immunol Res. 2021 Mar 8;2021:5599439. doi: 10.1155/2021/5599439 (PMC7959971; doi:10.1155/2021/5599439)
Supplement: Supplementary Materials — Supplemental Figure 1: clinical and immunological assessment of mice treated with NMU-23. Supplemental Figure 2: clinical and immunological assessment of NMU+/+ and NMU−/− CIA mice. Supplemental Table 1: the sequences of primers for quantitative RT-PCR. [file 5599439.f1.docx]

**Supplemental Figure 1.**

**
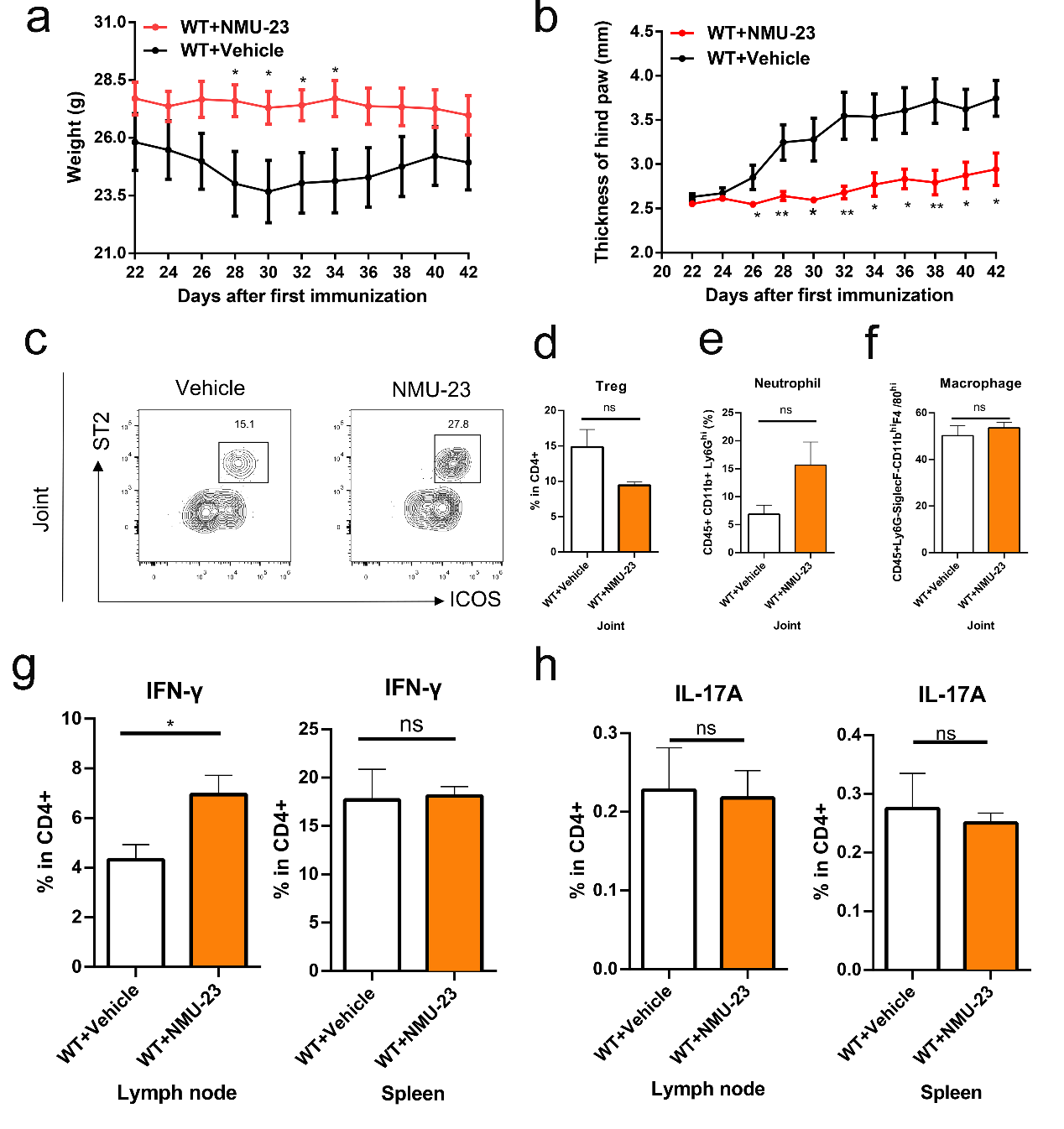
**

Supplemental Figure 1. Clinical and immunological assessment of mice treated with NMU-23. **a**-**b**, Body weight (**a**) and hind paw thickness determined every 2 days (n=8 each in two independent experiments); **c**, Representative flow cytometry plot of ILC2 in the joint of arthritic mice; **d**-**f**, The proportion of Treg (**d**), neutrophil (**e**) and macrophage (**f**) in the joint of arthritic mice; G-H, The percentage of Th1 (**g**) and Th17 cells (**h**) in mLN and spleen of arthritic mice. Data are shown as mean ± SEM (*P<0.05, **P<0.01 determined by Student’s t-test).

**Supplemental Figure 2.**

**
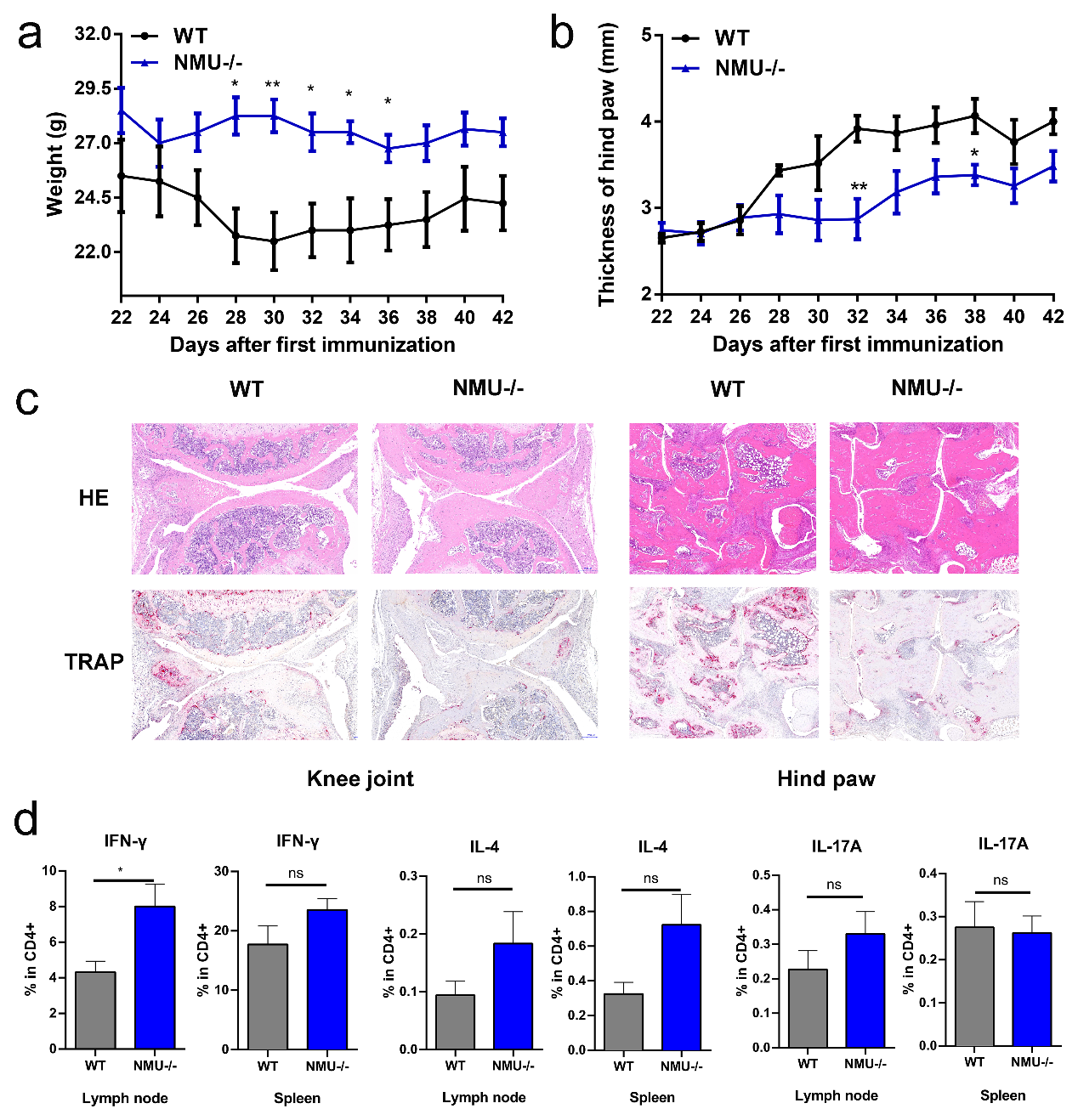
**

Supplemental Figure 2. Clinical and immunological assessment of WT and NMU^-/-^ CIA mice. **a**-**b**, Body weight (**a**) and hind paw thickness determined every 2 days (n=4 each in two independent experiments); **c**, Representative H&E and TRAP staining of the knee joint and hind paw of mice; **d**, The percentage of Th1, Th2 and Th17 cells in mLN and spleen of arthritic mice. Data are shown as mean ± SEM (**P*<0.05, ***P*<0.01 determined by Student’s t-test).

**Supplementary Table1.**

The sequences of primers for quantitative RT-PCR

| **Number** | **Gene** | **Forward (5’-3’)** | **Reverse (5’-3’)** |
| --- | --- | --- | --- |
| **1** | ***Gapdh*** | CATCACTGCCACCCAGAAGACTG | ATGCCAGTGAGCTTCCCGTTCAG |
| **2** | ***Gata3*** | CCTCTGGAGGAGGAACGCTAAT | GTTTCGGGTCTGGATGCCTTCT |
| **3** | ***Foxp3*** | CCTGGTTGTGAGAAGGTCTTCG | TGCTCCAGAGACTGCACCACTT |
| **4** | ***Il4*** | AGATCATCGGCATTTTGAACG | TTTGGCACATCCATCTCCG |
| **5** | ***Il5*** | CGCTCACCGAGCTCTGTTG | CCAATGCATAGCTGGTGATTTTT |
| **6** | ***Il13*** | GCTTATTGAGGAGCTGAG CAACA | GGCCAGGTCCACACTCCATA |
